# Seropositivity for *Enterocytozoon bieneusi*, Czech Republic

**DOI:** 10.3201/eid1602.090964

**Published:** 2010-02

**Authors:** Bohumil Sak, Zuzana Kučerová, Martin Kváč, Dana Květoňová, Michael Rost, Evan W. Secor

**Affiliations:** Biology Centre of the Academy of Sciences of the Czech Republic, České Budějovice, Czech Republic (B. Sak, M. Kváč, D. Květoňová); Centers for Disease Control and Prevention, Atlanta, Georgia, USA (Z. Kučerová, E.W. Secor); University of South Bohemia in České Budějovice, České Budějovice (M. Kváč, M. Rost)

**Keywords:** Enterocytozoon bieneusi, seropositivity, human, parasite, Czech Republic, dispatch

## Abstract

To determine seropositivity for *Enterocytozoon bieneusi* in the Czech Republic, we tested 115 serum samples from various groups. We found that 20% from HIV-positive persons, 33% from persons with occupational exposure to animals, and 10% from healthy persons were positive by indirect immunofluorescence assay. Proteins of 32 kDa were detected in serum samples from seropositive persons.

Microsporidia are small, single-celled, obligate intracellular parasites that were initially characterized as eukaryotic protozoa, but they have recently been reclassified as fungi. Since 1985, microsporidia have been identified as a cause of opportunistic infections associated with persistent diarrhea and weight loss in persons with AIDS (*1*). Because of heightened awareness and improved diagnostic methods, microsporidia infections have been recognized in a wide range of human populations, including organ transplant recipients, travelers, children, contact lens wearers, the elderly, and immunocompetent persons with no known risk factors (*2*).

Of the 14 species of microsporidia known to infect humans, *Enterocytozoon bieneusi* is the most common and is associated with diarrhea and systemic disease (*3*). Symptomatic *E. bieneusi* infections are primarily found in immunodeficient persons, although infection in immunocompetent populations is increasingly detected (*4*). It is unclear whether asymptomatic microsporidia infections persist in immunocompetent persons and can reactivate during conditions of immune compromise and are than able to be transmitted to others at risk, such as during pregnancy or through organ donation.

Studies focusing on risk factors associated with microsporidiosis will help define more clearly the sources of microsporidia that pose a risk for transmission in the environment so that preventive strategies can be implemented. To determine seropositivity for *E. bieneusi* in the Czech Republic, we used 2 serologic assays for detecting *E. bieneusi*–specific antibodies in serum specimens from HIV-positive and HIV-negative persons and from blood donors and persons with occupational exposure to animals.

## The Study

The National Institute of Public Health in Prague provided anonymous serum samples, originally collected for HIV diagnostics in 2007, from HIV-positive persons (n = 70) and healthy blood donors (n = 30). In addition, serum specimens from persons who worked with animals and animal excrement were collected after informed consent was obtained in 2007 (n = 15). Every specimen in the study was supplemented with data on the patient’s clinical symptoms (e.g., indigestion, abdominalgia). The study was approved by the Hospital České Budějovice, a.s. ethics committee (protocol no. 202/07). The serum specimens were frozen directly after recovery and were stored at –20°C. Patient identifiers were removed from the samples before testing.

*E. bieneusi* spores were purified from positive stool samples, originally obtained from an HIV/AIDS patient from Lima, Peru (provided by G.S. Visvesvara, Centers for Disease Control and Prevention, Atlanta, GA, USA), by using Percoll and cesium chloride gradient centrifugation as previously described (*5*). The spore suspension was stored in phosphate-buffered saline (PBS) supplemented with antimicrobial drugs at 4°C. The purity of spore suspension was tested by using light microscopy (optical brightener staining), and the background reactivity of serum specimens with bacteria was observed by using indirect immunofluorescence antibody (IFA) assay.

IFA was performed with purified whole *E. bieneusi* spores at a concentration of 10^5^/well. The serum samples were diluted in PBS by serial dilution, 1:10, 1:50, 1:100, 1:200, and 1:400, and results were compared with negative (1:100) and positive (1:400) control serum specimens. Serum specimens with titers >100 were considered positive on the basis of positive control serum titration. A total of 115 human serum samples were examined by IFA for antimicrosporidial immunoglobulin G. Specific antibodies against *E. bieneusi* were detected for 22 persons (19%; 95% confidence interval [CI] 12%–28%); 20% of HIV-positive persons (CI 11%–31%), 10% of blood donors (CI 2%–26%), and 33% of persons with animal risk exposure were positive (CI 11%–61%). CIs were calculated by the Clopper-Pearson formula for binominal counts (Table). None of the persons had demonstrated any clinical symptoms (e.g., loose stool, indigestion). The titers were higher (400) for HIV-positive persons and 1 animal keeper; the highest titer in blood donors was 200. No background reactivity was observed in tested serum samples with bacteria present in spore suspension.

**Table:** Seroprevalence of *Enterocytozoon bieneusi* in different groups, Czech Republic

Serum source	No. positive/no. examined (%)	95% confidence interval, %*	Maximum titer
HIV-positive persons	14/70 (20)	11–31	400
Blood donors	3/30 (10)	2–26	200
Persons with animal exposure	5/15 (33)	11–61	400
Total	22/115 (19)	12–28	400

*95% Clopper-Pearson confidence interval for binomial counts.

Proteins from 10^10^ purified spores were obtained by disruption of spores by using FastPrep 120 homogenizer and FastProtein Blue kit (both BIO-101, Inc., MP Biomedicals, Irvine, CA, USA) according to manufacturer’s instructions. Proteins were separated by using preparative 4%–20% acrylamide gradient Tris-HCl gel (Bio-Rad Laboratories, Hercules, CA, USA), electrotransferred onto nitrocellulose membranes (Schleicher and Schuell Bioscience, Inc., Keene, NH, USA), and cut into strips. Each strip was incubated with a 1:100 dilution of individual serum specimens in 0.3% Tween-PBS, peroxidase-conjugated goat antibody (Bio Source International Inc., Camarillo, CA, USA) diluted 1:4,000 in 0.05% Tween-PBS, and blots were developed by diaminobenzidine substrate solution containing H_2_O_2_. Although several different proteins were identified in specimens from seropositive persons, a parasite protein with a molecular weight of ≈32 kDa was predominant, this protein was not identified in any of the negative serum specimens (Figure). The results of immunoblot testing correlated with those of IFA; all serum samples with a titer >200 showed a strong reaction with immunodominant antigen in immunoblot.

**Figure:**
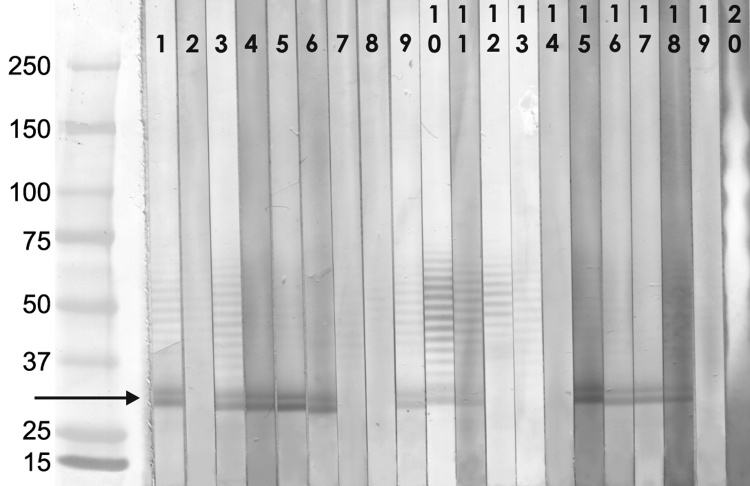
Western blot analysis of serum reactivity to *Enterocytozoon bieneusi* proteins, Czech Republic. Serum selection: HIV-positive persons (indirect fluorescence antibody [IFA] assay titers >400); blood donors, professionals with risk exposure (IFA titers >200). Serum samples diluted 1:100. Molecular weight markers (Precision Plus Protein Standard, Bio-Rad Laboratories, Hercules, CA, USA): lane 1, positive control (HIV/AIDS patient with proved *E. bieneusi* infection); lane 2, negative control (seronegative blood donor); lanes 3–8, selected samples from HIV-positive persons (3–6 IFA positive); lanes 9–14, selected samples from blood donors (9–11 IFA positive); lanes 15–20, selected samples from persons with occupational exposure to animals (15–18 IFA positive). Arrow indicates the 32-KDa protein fraction. Values on the left are in kilodaltons.

## Conclusions

The epidemiology of human *E. bieneusi* infection is poorly understood, and environmental factors that affect transmission of the organism have not been fully elucidated. Most reports addressing prevalence of microporidiosis are based on coprologic or PCR diagnostics, and the serologic screening of humans for microsporidia infection has mostly been limited to species that can be cultured in vitro (*6*–*8*).

Our survey was performed on a limited sample size from highly selected populations, which resulted in decreased statistical power. Although our findings are likely minimal estimates, given the uncertain duration of serologic response and <100% sensitivity of testing, they showed a 33% seroprevalence of *E. bieneusi* among animal keepers and 20% among HIV-positive persons. In studies in which infection was diagnosed by detection of *E. bieneusi* spores or DNA in stool, infection rates ranged between 1.4% and 78% (*9*–*12*). However, PCR and coprology are not able to discriminate *E. bieneusi* spores that have simply been consumed and passed through the intestinal tract from those resulting from active infection. In contrast, the detection of specific antibodies indicates that these persons experienced infection.

In the healthy population represented by normal blood donors, we detected a prevalence of only 10%, which is similar to previously reported prevalences (1.3%–8.0%) of *Encephalitozoon*-specific antibodies among HIV-negative persons such as blood donors, slaughterhouse workers, dog breeders, forestry workers, and pregnant women (*6*–*8*). In other studies, microsporidia infection of immunocompetent travelers with self-limiting diarrhea has been reported (*13*). The persistence of microsporidia despite resolution of the intestinal disorder suggests microsporidia infection may cause clinical signs (e.g., diarrhea) during the early stages of infection that resolve even though the microsporidia persist. In our study, the highest seroprevalence was in the group with professional exposures (33%), concurrent with a high titer of specific antibodies. Some of these professionals cared for pigs on farms, where *E. bieneusi* spores have been found in the feces of up to 94% of pigs (*14*). Other studies also confirm the possibility of occupational risk exposure to microsporidia spores. An immunocompetent laboratory worker occupationally exposed to *Encephalitozoon cuniculi* remained seropositive 38 months after treatment (*15*). These results indicate the possible role of animals as a zoonotic source of microsporidia spores and show a possible occupational risk for persons who work with animals and animal excrement.

Studies that focus on risk factors associated with microsporidiosis will more clearly define the environmental sources of microsporidia that pose a risk for transmission so that preventative strategies can be implemented. Because no data exist about latent infection in immunocompetent carriers, possible infection reactivation and person-to-person transmission risk through organ donation, our future studies will focus on detailed seroprevalence data among healthy populations, especially persons with occupational risk exposure, and will aim to elucidate the role of various animals in human infection. This information may lead to better identification of possible sources of microsporidial infections and help effect their prevention.
